# Quality of Life Philosophy I. Quality of Life, Happiness, and Meaning in Life

**DOI:** 10.1100/tsw.2003.102

**Published:** 2003-12-01

**Authors:** Søren Ventegodt, Niels Jørgen Andersen, Joav Merrick

**Affiliations:** The quality of Life Research Center, Copenhagen, Denmark

**Keywords:** quality of life, QOL, philosophy, human development, holistic medicine, public health, Denmark, meaning of life, purpose of life, life mission, happiness, SEQOL

## Abstract

In the Danish Quality of Life Survey, we asked 10,000 people about their quality of life with the validated SEQOL questionnaire with more than 300 questions on their quality of life. How did they feel? How content were they with their lives? How happy were they? Did they feel their needs were fulfilled? And many more questions. We asked the questions we believed to be important for their quality of life (QOL). The results were quite surprising and forced us to recontemplate the following philosophical questions: What is quality of life, happiness, and meaning in life? What is a human being? Do we need a new biology? Is the brain the seat of consciousness? How do we seize the meaning of life and by doing so, will we become well again? What are the key concepts of quality of life? The meaning of life is connectedness and development. It is about realizing every opportunity and potential in one’s existence. The opportunities must be found and acknowledged. What do you find when you find yourself deep down? You find your real self and your purpose in life. You realize that you are already a part of a larger totality. Antonovsky called it “coherence”. Maslow called it “transcendence”. Frankl called it “meaning of life”. We call it simply “being”.To test if these philosophical questions are actually relevant for medicine, we looked at the consequences for patients being taught the quality of life philosophy. Quite surprisingly we learned from our pilot studies with “quality of life as medicine” that just by assimilating the basic concepts of the quality of life philosophy presented in this series of papers, patients felt better and saw their lives as more meaningful. The improvement of the patient’s personal philosophy of life seems to be the essence of holistic medicine, helping the patient to assume more responsibility for his or her own existence.

## INTRODUCTION

Through all ages, philosophers have been occupied with the immense questions and seemingly unsolvable problems related to the quality of human life. For half a century, grand thinkers have been reflecting on the meaning of life and how to improve it and quite surprisingly, a sign of our time is that these problems are starting to soften up on the thinkers[1,2,3]. For more than a decade, the Quality of Life Research Center in Copenhagen, Denmark and its scientific coworkers (mostly in Israel and Norway), have also walked this path, publishing books[4,5,6,7,8,9,10,11] and scientific papers[12,13,14,15,16,17,18,19,20,21,22,23,24,25,26,27,28,29] on the good life and the good work. Except for two books[4,5] and one paper[12], our works have often discussed philosophy related to the concept of quality of life (QOL) and quality of working life (QWL), but have not addressed the philosophical questions directly.

But as philosophy is the basis of science, this is not wise. We need an explicit discussion of the philosophy behind the major aims and developments in our medical science, especially as medicine is not merely a natural science, but as much a humanistic science, embracing difficult subjects like consciousness, happiness, and meaning of life.

In our series of papers on quality of life philosophy, we take our offset in the Danish Quality of Life Survey, where we asked 10,000 people about their quality of life with the validated SEQOL questionnaire[16,17] with more than 300 questions on their quality of life. How did they feel? How content were they with their lives? How happy were they? Did they feel their needs were fulfilled? And many more questions. We asked the questions we believed to be important for quality of life. The results[8,9,10,11,22,23,24,25,26,27] forced us to contemplate deeply on the following philosophical questions, which are expressed in the titles of the six papers in this series:

- Quality of Life Philosophy I. Quality of Life, Happiness, and Meaning in Life
- Quality of Life Philosophy II. What is a Human Being?
- Quality of Life Philosophy III. Towards a New Biology: Understanding the Biological Connection between Quality of Life, Disease, and Healing
- Quality of Life Philosophy IV. The Brain and Consciousness
- Quality of Life Philosophy V. Seizing the Meaning of Life and Becoming Well Again
- Quality of Life Philosophy VI. The Concepts

To argue that these philosophical questions are actually related to medicine, we need to look at the consequences of patients being taught this philosophy as discussed here. Quite surprisingly, it seems that just assimilating the quality of life philosophy already makes things feel better and more meaningful. We call this "quality of life as medicine"[28,29], and this improvement of the patient's personal philosophy of life seems to be the essence of holistic medicine, helping the patient to assume responsibility for his or her own life[30].

The quality of our reflections is soft and general — pretty vague, if you are accustomed to the "hard" quality of natural science — as much philosophy is. Nevertheless we find such general perspectives of utmost importance as all science is built on such general considerations of values, perspectives on reality, interpretations of life, and preferences of focus. So please bear with this vagueness, and look for the abstract, quality of life–supportive perspective, which materializes in our papers on quality of life philosophy, as we believe that such a positive interpretation of life and reality can help many patients.

## STARTING TO WONDER

Studying the results from the Danish Quality of Life Population Survey, a very clear and somewhat surprising picture emerged. During the same period, other researchers found similar results and had similar reflections[31,32,33,34,35,36,37,38,39,40,41,42,43,44].

The essence of what we found was this: Executives do not feel substantially better about their lives than the people on the shop floor, people who work with knowledge on the highest professional level do not feel better than unskilled workers, or in simple terms the rich did not feel better than the poor.

Apparently, money, power, or learning was not the most important aspect of the good life. Oddly enough, it is exactly these things people in our culture run after like crazy. How come? How has it come to be that money has become a value in itself? Something worth striving for in and of itself? Has everybody gone mad?

Let us ask what is it, all in all, that gives us the experience of a good life? According to our study, some of the most important factors were: How we relate to ourselves, how we relate to other people, and the extent to which we do something we really like. Expecting these three things from life appears obvious, almost commonplace, but in reality they are very hard to get.

Recall one of those rare occasions when you spoke confidentially with your best friend. You were having a nice dinner with a good bottle of wine, and you told the friend about your life, relationship, work, friends, all your dreams. Suddenly you may have experienced great sorrow or perhaps even began to cry. Life did not quite turn out the way you dreamt about. Somehow life must have more in store for us. Is this all there is for us?

Life is often difficult. The senses and feelings carry us away: What others expect from us, all that we have learned about being sensible, inhibits and pressures us. The reality can easily seem to be one chaotic stream in which you are caught. Or you can experience the world as being hard as granite: You cannot scratch it with a nail, you cannot make other people change, you cannot make any difference at all. When you are low and without energy you are also often unlucky and not able to understand what it is all about, and what you can do to get on in life.

Life as an entity, the entire existence when all circumstances are considered, can be perceived as good or bad, sometimes better and sometimes worse. There are many ways to evaluate life. When taking stock, it is usual to compare yourself with your neighbor. Is my life better or worse than his or her life? You can work your way through life systematically and look at your health, economy, partnership, friends, family, and your working situation. Also, you can just sense if you feel better, are more on top of things, and have more energy. When you compare yourself with your neighbor, it is normally not that hard to conclude that you do have a decent life.

It is far worse when you look deep within yourself, i.e., you can use your innermost dreams about a good life as comparison. Do I have the life I really wanted, deep down? Is my partner the love of my life? Are my friends sincere and intimate friends with whom I can share everything and whom I can really trust throughout my lifetime? People who want the very best for me? Is my relationship with my children, my parents, my siblings, and the rest of the family truly as good as it could be?

Is my work as interesting as I dreamt it to be? Do I do what I really want to do? Do I continue to improve myself or have I stopped my development? Do I genuinely use my skills at work, expand, and prove useful in the world? Do I profoundly make a contribution in order to make a better world? Does my life make a little difference here on Earth?

## YOUR INNERMOST DREAMS ABOUT LIFE

When we begin to dream the great dreams of life — or perhaps when we remember the dreams we had in our youth, just before we turned adult — it is clear that the life we live at the moment is often a far cry from the dreams and the hopes of our youth.

"I have been so naive" one thinks, "at that time I believed that the great love could last forever. Well, now I know that a love affair fades away and life together inevitably turns into routine, when you know each other intimately. We have been together for five years now, and I cannot expect it still to be new and interesting."

One may speculate that such thoughts contain numerous lies and self-deceptions that indeed make us accept the sorry mess we sometimes are in. Do we really know ourselves at all? Is life not a profound mystery? Do we not continue to discover new and unexpected dimensions of ourselves that we can use to grow and blossom? Can we not keep our hearts warm, even when things are changing? Should we not consider our dreams precious and refuse to give up? Is it not foolish of us and completely wrong to sell out our dreams, even though making them come true turns out to be harder than we thought?

Some people have self-confidence. They seem to handle everything in style and what they do not know how to do, they quickly learn. Such people are almost unbearable. When we struggle with life and have all sorts of problems, we see these people being successful again and again.

They plunge into new and impossible tasks and projects. They are often roughly handled, but it does not seem to bother them. After a certain number of attempts, they usually succeed. Things turn out as they want them to. The rest of us feel like quitting when the things we are working on keep failing. It is hard to understand what drives these people to persevere when others seem to give up. What kind of force makes them keep going?

It is obvious that people with genuine self-confidence somehow have luck on their side. The unceasing efforts, which help you learn all the time and improve your next attempt, will lead to triumph eventually. A steady flow of successes gives the self-confidence that is required for you not to give up. But where does this energetic persistence come from? What is the difference between the person who gives up and the winner?

We believe that people who fight the struggle of their life and gladly take the existential challenge upon themselves really want a better life. The meaning of their life is obvious — they know what they want and fight to reach their goal.

In reality it might be put as simply as that. Life is about being aware of what you want, to know your innermost dreams, to know the opportunities around you, which means to know the world you are living in, and to have the values that make it possible to unite your dreams with the opportunities that reality presents[45].

In this way, life is about building a bridge between the deep existence and the world around us[46]. The meaning of life is to create a connection between our inner depths and the outer world. It is about finding the dreams and all the hidden potentials and fighting to bring them out. This is the idea of quality of life as life self-realization[2].

## OUR MAP OF REALITY

Our brains help us build a bridge between the inner individual and the world around us[47]. The brain holds a mental map of the world: A very detailed description of the world around us, of ourselves as persons, and of the life inside us.

Some examples of what we know about the world around us: We know specifically that a red light means stop, "a" is the first letter in the alphabet, coffee is drunk from cups, and metal that glows red is extremely hot and should not be touched. What we know about ourselves includes the all too common self-assessment deriving from hearing the words "Just who do you think you are?" too often. "Oh, well, I am nothing special, I am not sexually attractive, my looks are nothing to write home about, I am not particularly smart." What we know about the life inside us includes the meanings of numerous inner sensations — some physical (such as fatigue, desire, or pain) and others more emotional, such as a feeling that something is valuable, meaningful, or true.

In our view, the quality of our mental map of reality is of the utmost importance for our quality of life.

When we sense — see and hear — the world around us, we interpret it by means of this mental map. We filter, so to speak, all the impressions through overdescription of the world and we end up being fairly well adapted to it. We are pretty good at sensing, thinking, and acting. The world appears very familiar to us. Apart from the odd detail that becomes obsolete and has to be corrected now and then, we tend to believe more and more firmly throughout life that the description of the world that our parents and our teachers taught us is correct.

This happens while we act in the world on our own and experience again and again that the map is, in fact, true. The few details we change in our parents' map, so as to make it more truly our own, become an important part of our own identity and thus ends up being disproportionately prominent. In general terms, the fairytales of childhood become the reality of adulthood.

There are many terms for this mental map: philosophy of life, self-image, worldview[48], weltanschauung[49], ontology[50] — all terms for very much the same thing. The map contains a description of our view of inner life, the outer world, and our self-image in between.

The point of having an awareness of reality is clear enough: We need it to realize our dreams and wishes more efficiently. It gives us an opportunity to navigate reality and act single-mindedly and with great effect. Because we are conscious of the world we can change it. Unfortunately, we have not yet harnessed our amazing effectiveness — as when the entire rain forest in Brazil may be cut down 20 years from now, thanks to greediness, irresponsibility, and the invention of 20-ft chainsaws.

The detailed description of the world that our mental maps represent gives us serious problems, because you can be lost in your own description of reality. For example, your mental map says that the important things in your life are x, y, and z. Then you fall seriously ill. You may discover that other things in life are far more important. You come to the painful realization that you have lived your life on a lie. One of our biggest problems is that we become experts at lying to ourselves, because we are free to describe reality any way we want.

Our inherited descriptions of the world constantly confirm themselves. For example, some people are raised with little love. Bitter experience has taught them that other people are neither loving nor trustworthy. Understandably enough, they treat other people without love and trust, whereupon they discover that people are not loving or trustworthy. The pieces fit. What we have learned is perfectly true. Nevertheless, there is little truth in it, because with another set of assumptions about what to expect from other people, one's view of the world and one's behavior would be much different. And one's quality of life would improve.

## A PHILOSOPHY OF LIFE WITH FOCUS ON THE HUMAN BEING

How we experience life is a direct result of our mental map. By nature we are free to describe the world in an amazing variety of ways, but once our parents and our culture have chosen a description, we are forced to live accordingly. Everything in life adjusts itself to this description. Life as a whole is tinted with a certain color. Grey and dull, black and sinister, or happy and colorful, depending which chord is struck in the mental map.

People who learn to believe in themselves seem to be wiser than people who have little faith in themselves. If the philosophy of life does not allow the individual any special opportunity to solve problems and create value in her or his own life, the person is caught in a trap, unable to tap the wisdom in the depths of life[51].

People who do not believe that you can follow your heart and live in harmony with yourself and hold an inner truth about life will never be able to listen for this inner wisdom[52]. People's philosophy of life makes them open to life, or close them up.

Large parts of Denmark's healthcare and welfare systems are based on a philosophy of life that does not hold much room for — let alone believe in — the individual's own wisdom of life and capacity for solving his or her own problems and making the right decisions. Keeping the tradition, medicine regards a human being as an ingenious machine that physicians are able to repair with little interference from the patient. Generally, the medical profession does not believe much in the causality of consciousness, in spiritual dimensions of existence, or in that people can help themselves get well to any appreciable degree. There is very little faith in the power of the individual over his or her own existence.

An alternative philosophy of life is needed, where every change starts with the individual person, meaning that a sick person is able to do much more for him- or herself than any physician. A lonely person can do more for himself than any psychologist. A person with sexual problems can help himself to a greater extent than any sexual therapist. Problems with bringing up children are far better solved through a personal, independent, and goal-directed effort than by handing over the responsibility to educators and child psychiatrists. It is far healthier to reflect on what is good for you to eat than to be guided by a dietician.

The truth probably lies in between these views. In principle, we are personally in favor of the latter view, but of course this does not mean that there is no need for physicians or psychologists anymore, merely that their roles should be different from what they are now. The world is complicated and we need profound and certain information from experts, but the experts have to stop being autocratic authorities and become more humble consultants that respect the fact that ultimately, we know as individuals what is right and wrong for us.

## TAKING RESPONSIBILITY FOR YOUR LIFE

When you have lived a bad life, full of insolvable problems and great difficulties and without any real happiness or meaning, what you need is something rather peculiar: To pull yourself up by your bootstraps.

Naturally, this is not about defying the laws of nature. It requires acknowledging that life holds wisdom that is not currently part of one's mental map. In order to open yourself up for life, to place yourself in the power of life, it is necessary to ask yourself: What is it that I have not yet understood about life? What do I have to believe and recognize beyond any reason and experience, if I love life?

It is necessary to demand of yourself that you understand why your life is not filled with opportunity, freedom, love, and meaning. That is the beginning. You must try to fathom your deeper existence, your heart, or soul. You may well entertain abstract philosophies, but you can easily end up philosophizing the rest of your life.

However, revising your view of life means to take responsibility. This is not a rational process; rather, you recognize that life with its immense complexity cannot be figured out. As a consequence, you have to stop to observe and analyze things mentally and start a new life as a participant. A good life is about acting, being part of, being a participant in life. It is about closeness and direct understanding through the meeting, the confrontation, the touch, and the melting together.

The hallmark of life is communication[53]. The cells in our body communicate so efficiently that the distance between them practically disappears and they melt together to what we know as ourselves: "me". For example, when we have a partner whom we love deeply, we may experience that the distance disappears and you melt together as one. In a close moment, you understand each other fully. It is this closeness that love is all about. This is what ought to fill life in all its relations, in the relationship to the partner, the friends, the children, the work — in order to make life genuinely good.

## QUALITY OF LIFE IS MORE THAN SATISFACTION

If asked how you feel, you may answer “good” or “bad”. Inspired by the Danish psychiatrist Anton Aggernæs, we have called this part of quality of life “feeling good”. When life is not genuinely good — which is often the case — there are many tricks you can use to save appearances. Superficially, you can live your life better than it is, but at a deeper level, this lie does nothing to make things better.

If you happen to get your spinal cord ripped over in a traffic accident, you will most likely be paralyzed in your arms and legs for the rest of your life. You may only be able to play computer games with your nose — and most of us would rather die than end up in this situation — but surprisingly enough, it turns out that there are many people who live stable lives in this way and after 5–10 years are often content with their existence.

In general terms, any kind of difficult situation that stabilizes and remains so for some years — whether chronic illness, prostitution, or extreme poverty — in time becomes a habit. Likewise we can adjust ourselves to almost anything, as long as the situation is stable and a sufficient period of time passes by.

Our capability to adjust ourselves is extremely high. We can become content under basically any kind of circumstances, if only we live a stable life in them for a long time, without any chances of change. The quality of life that you achieve by adjustment is called contentment. It is a subjective (experienced, inner) dimension of quality of life, which in reality does not hold much about the real quality in life, below the surface. The contentment is mental, in the mind: When we dream our dreams small enough, to make them fit our pitiful life, we become content.

In this view, contentment is a trap that we fall into all too easily. Once you have become content and well adjusted, the driving force that could have propelled you and made you fight for a better life has completely vanished. Contentment is relevant to the quality of life, but only in a minor way. The “quality of life” is a kind of umbrella description that encompasses many ways that life may appear good or bad. There are profound and superficial ways to feel good. Some involve seeing life from the inside, some from the outside. Some involve seeing your existence very locally, some regard life as a whole.

Contentment, for example, is something superficial, it is life regarded from the inside (subjectively contrary to life regarded from the outside: objectively), and it is something local (because it is mental, in your mind). Other ways to look upon life locally are sensual pleasures, such as eating (it feels good in the mouth). Some people are able to divide their bodies and minds so efficiently that the delight of eating can continue long after the body has fulfilled its need, leading to obesity. Other people are obsessed by sex. Their efforts to find sexual pleasure distort their lives, especially when combined with an inability to hold on to life as whole. The worse you feel inside, in your heart, or in total, the more tempting it is to give yourself away to more superficial and local pleasures now and then[54].

## HAPPINESS AND THE FULFILLMENT OF NEEDS

Happiness is another and more prominent aspect of the quality of life. Happiness involves our entire existence. When we are happy, every fiber in us hums to the right tune. Happy people are radiant. We become happy when our life succeeds. Happiness is about our visions and big dreams coming true. You do not get truly happy by getting another stamp for your fine stamp collection.

Yet another aspect of the quality of life is covered by the concept of needs, even though they overlap each other to some extent. We have needs for certain things — friendship, for example — but we do not need stamps in the same way. The stamps are an example of something we are rather free to choose as one of our values, but it is hardly a basic need. Of course, there may be stamp collectors who live extraordinarily good lives because the stamps have become a good opportunity to share and have fun with other people. In a closer analysis, it often turns out that the urge to collect stamps is just an expression of an enormous surplus of energy by this person, and that his or her quality of life does not come from the stamps per se.

A better example than the stamps may be money. A lot of people build their entire existence on acquiring money. In our population study, we found no association between annual income and happiness in the Danish population. On the average, people earning USD/Euro 8,000 per year are precisely as happy as people earning more than USD/Euro 70,000 per year. Thus, money is an example of something you may choose to strive for, but which will only give you very little pay-off in existential terms.

We are rather free to choose our own external or superficial values, but we are not that free to choose our internal or deeper values. Happiness results only when our chosen values are in accordance with our heart or soul, with our deeper natures as human beings. The American psychologist Abraham Maslow[2] concluded in the 1960s that the human has four basic needs: needs related to the body (heat, food, sex, shelter), social needs for genuine relations to others, needs for being useful, and finally a need to "actualize" oneself. Maslow was quite pessimistic about the fourth need, which he thought merely three or four people in the world had managed to fulfill. Therefore, the idea of a hierarchy or a pyramid of needs was introduced, in which you start with the bodily needs and end up with the need for self-actualization. Later on problems with the pyramid arose. Hungry children also play!

Because there are so many conceptions of human needs, and because these conceptions are culturally bound, is it rather difficult to determine what needs a person actually has. Thus, the concept of needs has been criticized by many scholars for being diffuse, unsuitable for research, and as material for personal development. The realization of our profound potentials, which we also call self-realization, appears to us as a more precise and useful concept.

## SELF-REALIZATION

"Self-realization" covers deeper and more comprehensive aspects of the quality of life[2]. To us, it is more fundamental and closer to the soul than the notion of needs fulfillment. It says more precisely that life is about the creation and realization of something that issues from a person's innermost depth — the heart or the essence of existence.

The number one enemy of self-realization is resignation or the process in which we dream our dreams smaller as we downsize our ambitions, thanks to hundreds of minor and major lies about the values in life. Most adults wear a whole woolen sweater knitted by these many lies. It is effectively an armor that keeps us from recognizing and sensing our innermost soul. Any symptom, from headaches to financial problems, can be seen as the price you pay for the distance the sweater keeps between you and your soul. But somehow the sweater is never perfect, there are always loose ends to the yarn that the sweater is made from. Always little holes from which frustrated life dreams and deeper imbalances will emerge, sometimes as a dull, nagging, existential pain, at other times as distinct symptoms of something that is wrong in your life. If you have the energy, you could unravel the whole sweater of lies. In your efforts to do so, you have to ask yourself: Why? When you get the answer to that question, you could ask again: What is the reason behind it? (For an example: Why do I have a headache? Because I just had an argument with my wife. Why did I argue with my wife? Because I wanted her do as I told her. Why did I want that? Because it is easier to change her than to change myself. Why do I resist changing myself? Because I feel that I am going to die if I begin to let go of my stubborn ego.)

Again and again you could unravel, until the whole set of self-deceptions, illusions, and misunderstandings are revealed before your eyes. Slowly you may begin to realize that life is much greater and more mysterious than you thought and that you are facing far bigger problems within yourself than you imagined[50].

## THE MEANING OF LIFE

If we descend even deeper into existence, we reach the deepest level that we can verbalize[55]. Deep down in the core of existence, we find the very recipe for being a human. It has evolved from the time when life started in the primordial soup on Earth about 3 billion years ago, and it is still evolving, in a flow that is faster than ever.

To us, the meaning of life is about placing yourself in a direct connection with that flow. About giving yourself totally to life and serving the great powers in the world, the powers that create, transform, and restore our world. We believe there is an underlying order, an intelligent or creative process that exerts pressure to create and renew everything that exists. There is also something even deeper, something spiritual at play. This brings us close to the point where words tend to be inadequate. Probably you cannot go any deeper with language. You may rely on art and express yourself through poetry or music. Artistic creativity certainly expresses the very intelligence of life, which manifests itself directly, beyond the bounds of limited reason.

One way to an understanding of the profound things in life could be through biology. The powers that operate in living matter and drive biological development and evolution are quite peculiar — and apparently far more complex than Charles Darwin suggested in *The Origin of Species*[56], namely natural selection through the struggle for survival. In a later paper we will address life and biology more directly. For now, let us just mention that our dreams (whether dreamt during the day or the night) about a better life seem to be under the influence of gigantic powers, which in the end work for the further development of humanity as a whole, by means of some profound coexistence or an subconscious community between all people.

The evolutionary development of the human being is presumably not finished at all, but may continue for thousands of years into the future. Through the realization of one's deepest dreams, every person can unfold his or her own life in an extremely creative way and thereby also contribute to humankind's great developmental project. The idea that you contribute to the development of humankind as you grow personally makes the fact that personal growth is so cumbersome seem more reasonable.

## CONCLUSION: THE CORE OF LIFE

Even if it is not possible to articulate the deepest dimensions in life (and therefore not possible to measure them in spite of our best research efforts), we somehow have to find a way into the core in life if we want to live a good life. We have to open ourselves to a wordless, intuitive, but intensively meaningful part of life that is often denied by our rational part.

We need to access the core of life itself, the wildness or the madness that lies just beneath the skin and which longs to be set free and express itself. We do not mean crude and uncontrollable drives, but we have to find the fire within, find the commitment and presence of mind to be in intense exchange with our reality, within and without.

We have to give everything we have got and be able to take what the world has in store for us. This may sound a bit starry-eyed, as something we might try to do during a vacation in Bali, but which is hard to fit into our everyday lives at the office. But if we talk about professional development, for example, this is exactly a process in which we try to master our job so well that we can express ourselves fluently, untroubled and spontaneously. Life's peaks are where we truly use our resources to the full and give ourselves completely to what we do, where the barriers disappear and we become one with our activity.

Life does not get any better than this: Where we forget ourselves and live with our entire heart, our entire existence. Life flows in intense, new forms and patterns that are pregnant with meaning. You know that what you are doing is precisely what you were put on Earth to do. This experience is the full and complete life.

The meaning of life is connectedness and development. It is about realizing every opportunity and potential in one's existence. And the opportunities have to be found and acknowledged. What is it you find when you find yourself deepest down? Your belly button? An island disconnected from others? No, you find connection. You find your real self and your purpose in life[12]. You realize that you are already a part of a larger totality. Antonovsky called it coherence[1]. Maslow called it transcendence[2]. Frankl called it meaning[3]. We call it simply being.

## ACKNOWLEDGMENTS

This study was supported by grants from The 1991 Pharmacy Foundation, as well as by supplementary grants from Goodwill-fonden, the JL-Foundation, E. Danielsen and Wife's Foundation, Emmerick Meyer's Trust, the Frimodt-Heineken Foundation, the Hede Nielsen Family Foundation, Petrus Andersens Fond, Wholesaler C.P. Frederiksens Study Trust, Else & Mogens Wedell-Wedellsborg's Foundation and IMK Almene Fond. We gratefully acknowledge the critical scrutiny and expert linguistic assistance of Ib Ravn, Ph.D. and also the assistance of the philosopher Maximilian Kroman. The research was approved by the Copenhagen Scientific Ethical Committee under number (KF)V.100.2123/91.

## BIOSKETCHES

**Søren Ventegodt, MD,** is the Director of the Quality of Life Research Center in Copenhagen, Denmark. He is also responsible for a Research Clinic for Holistic Medicine in Copenhagen and is a popular speaker throughout Scandinavia. He has published numerous scientific or popular articles and a number of books on holistic medicine, quality of life, and quality of working life. His most important scientific contributions are the comprehensive SEQOL questionnaire, the very short QoL5 questionnaire, the integrated QOL theory, the holistic process theory, the life mission theory, and the Danish Quality of Life Research Survey, 1991–94 in cooperation with the University Hospital of Copenhagen and the late pediatric professor Bengt Zachau-Christiansen. E-mail: ventegodt@livskvalitet.org. Website: www.livskvalitet.org/

**Niels Jørgen Andersen, MSc,** Professor, Department of Innovation and Economic Organization, Norwegian School of Management. This department conducts research and provides teaching in central topics related to innovation, business development, management of global companies, business history, and economic organization. Research activities within the Department are related to four core subjects within the discipline: business history, cooperative organizations, business development and entrepreneurship, and finally studies of industries with a special focus on the electricity industry. He is also the dynamic chairman of the nonprofit organization Stiftelsen Holistisk Medisin Scandinavia, which aims to support the scientific development, research, and documentation of complementary and holistic medicine in Scandinavia. E-mail: niels.j.andersen@bi.no. Website: www.bi.no/users/fgl93013/

**Joav Merrick, MD, DMSc,** is Professor of Child Health and Human Development affiliated with the Zusman Child Development Center and Division of Community Health at the Ben Gurion University, Beer-Sheva, Israel and presently the Medical Director of the Division for Mental Retardation, Ministry of Social Affairs, Jerusalem and the Director of the National Institute of Child Health and Human Development. He has numerous publications in the field of child and human development, rehabilitation, intellectual disability, disability, health, welfare, abuse, advocacy and prevention. Dr. Merrick received the Peter Sabroe Child Award for outstanding work on behalf of Danish Children in 1985 and the International LEGO-Prize ("The Children's Nobel Prize") for an extraordinary contribution towards improvement in child welfare and well being in 1987. E-mail: jmerrick@internet-zahav.net. Website: www.nichd-israel.com
